# Elevating Ascorbate in Arabidopsis Stimulates the Production of Abscisic Acid, Phaseic Acid, and to a Lesser Extent Auxin (IAA) and Jasmonates, Resulting in Increased Expression of *DHAR1* and Multiple Transcription Factors Associated with Abiotic Stress Tolerance

**DOI:** 10.3390/ijms22136743

**Published:** 2021-06-23

**Authors:** Sean M. Bulley, Janine M. Cooney, William Laing

**Affiliations:** 1 The New Zealand Institute for Plant and Food Research Limited, Te Puke 3182, New Zealand; 2 The New Zealand Institute for Plant and Food Research Limited, Ruakura, Hamilton 3214, New Zealand; janine.cooney@plantandfood.co.nz; 3 The New Zealand Institute for Plant and Food Research Limited, Palmerston North 4410, New Zealand; William.laing@plantandfood.co.nz

**Keywords:** ascorbate (AsA), dehydroascorbate reductase (DHAR), abiotic stress, 9-cis-epoxycarotenoid dioxygenase 3 (*NCED3*), abscisic acid (ABA), phaseic acid (PA)

## Abstract

Gene expression and phytohormone contents were measured in response to elevating ascorbate in the absence of other confounding stimuli such as high light and abiotic stresses. Young Arabidopsis plants were treated with 25 mM solutions of l-galactose pathway intermediates l-galactose (l-gal) or l-galactono-1,4-lactone (l-galL), as well as L-ascorbic acid (AsA), with 25 mM glucose used as control. Feeding increased rosette AsA 2- to 4-fold but there was little change in AsA biosynthetic gene transcripts. Of the ascorbate recycling genes, only *Dehydroascorbate reductase 1* expression was increased. Some known regulatory genes displayed increased expression and included *ANAC019*, *ANAC072*, *ATHB12*, *ZAT10* and *ZAT12*. Investigation of the *ANAC019*/*ANAC072*/*ATHB12* gene regulatory network revealed a high proportion of ABA regulated genes. Measurement of a subset of jasmonate, ABA, auxin (IAA) and salicylic acid compounds revealed consistent increases in ABA (up to 4.2-fold) and phaseic acid (PA; up to 5-fold), and less consistently certain jasmonates, IAA, but no change in salicylic acid levels. Increased ABA is likely due to increased transcripts for the ABA biosynthetic gene *NCED3*. There were also smaller increases in transcripts for transcription factors *ATHB7*, *ERD1*, and *ABF3*. These results provide insights into how increasing AsA content can mediate increased abiotic stress tolerance.

## 1. Introduction

l-ascorbic acid (AsA) is an essential dietary nutrient (vitamin C) which humans cannot synthesize due to mutations within the *l-gulono-g-lactone oxidase* (*GLO*) gene [1] (GLO catalyzes the last step in vitamin C biosynthesis), therefore elevating AsA in plant foods for human nutrition is of interest to plant breeding [2]. For plants, AsA is an important primary metabolite protecting photosynthesis and supporting enzyme action, and elevating AsA is also of interest to improve plant resilience and stress tolerance [3]. One action of AsA in plants is neutralizing reactive oxygen species (ROS; chemically reactive chemical species containing oxygen, such as peroxides, superoxide, hydroxyl radical, singlet oxygen, and alpha-oxygen), sometimes through direct interaction, but in the case of H_2_O_2_ it is through it being a co-substrate for ascorbate peroxidases. AsA also participates in signaling pathways initiated by both ROS and reactive nitrogen species [4]. Its reducing activity also protects other anti-oxidants such as Vitamin E (tocopherol) [5] and violaxanthin, with AsA being a co-factor of violaxanthin de-epoxidase [6]. Its pro-oxidant activity in the apoplast to rapidly hyperpolarize the plasmalemma and/or the generation of highly reactive •OH radicals has been suggested to be involved in cell wall loosening [7,8,9,10], promoting cell expansion and/or cell wall weakening (e.g., fruit softening). AsA is either a co-substrate or stimulant of many enzymes, a notable example of the former being 1-aminocyclopropane-1-carboxylate (ACC) oxidase, the enzyme that produces the hormone ethylene [11]. These typically have metal cores (e.g., Fe or Cu) which require maintenance in a divalent state to enable enzyme action. A major group of AsA requiring enzymes are the diverse 2-oxoglutarate-dependent dioxygenase class of enzymes which number in the thousands, and which ACC oxidase belongs to [12,13,14]. This means that changes in AsA can have pleiotropic effects by altering the activities of multiple enzymes. In animals AsA is involved in epigenetic regulation, via demethylation of nucleotides and histones [15]. This has been established in mammals through the identification of Ten-Eleven-Translocation (TET) enzymes [16,17,18], which are 2-oxoglutarate- and AsA-dependent dioxygenases. It is unknown if there are similarly functioning enzymes in plants.

Biosynthesis of AsA is intimately linked with the mitochondrial electron transport chain through l-galactono-1,4-lactone dehydrogenase (GLDH) [19,20,21,22,23,24,25], enzyme action, and also through the role of GLDH in the assembly of complex I holo complex [26]. This may play a role in balancing electron flows under different environmental circumstances [27].

**Figure 1:**
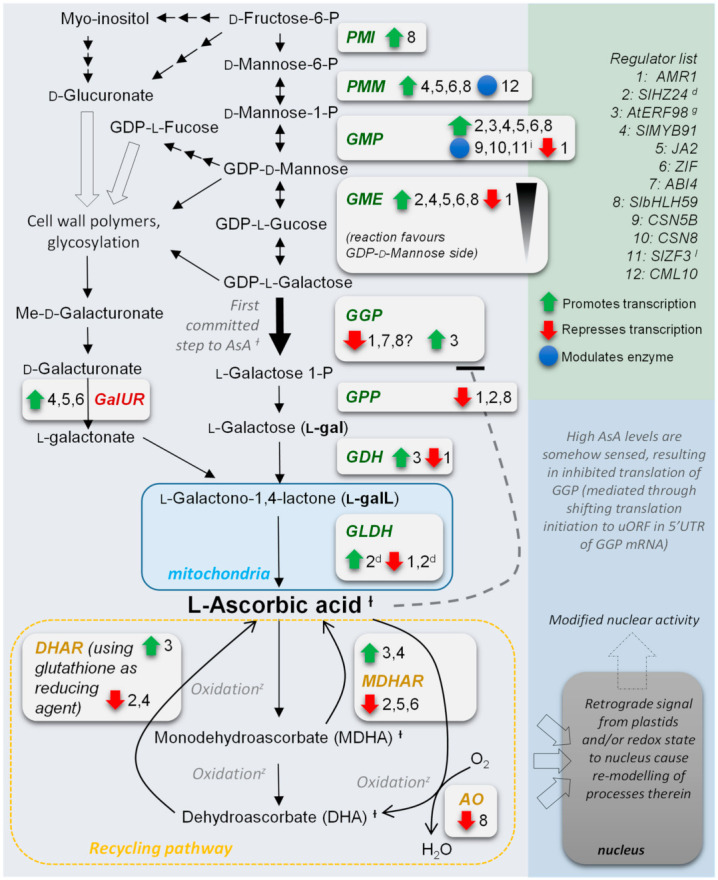
Overview of main route to ascorbate through l-galactose in plants highlighting various features and known regulatory points of control for the various enzymatic steps. Alternative pathways are included but not detailed. See text for enzyme abbreviations (isoforms not listed e.g., VTC2/VTC5 etc.); red text denotes Galacturonate pathway enzyme; green text denotes l-galactose pathway enzymes; and dark yellow text denotes recycling pathway enzymes. Triplet arrows denote two or more steps of conversion. Hollow arrows denote substrates shunted to other processes/states (e.g., polymerization). Footnotes: ^ɫ^: collective noun for these three oxidative states is ‘ascorbate’, also expressed as ‘total ascorbate’ in empirical measurements; ^Z^: oxidation can be by direct chemical interaction or enzyme mediated (e.g., by ascorbate oxidases/peroxidases etc.); ^d^: different action between tissue types e.g., leaves versus fruit. ^g^: also activates *MIOX4* (involved in myo-inositol synthesis) transcription (not detailed). ^i^: binds to CSN5B to inhibit CSN5B binding to GMP.

AsA varies by tissue, developmental stage, and environmental conditions. The regulation of ascorbate levels in plants has only relatively recently become better understood. The enzymes acting in the main biosynthetic pathway in Arabidopsis through l-galactose (Smirnoff-Wheeler pathway [28]) have been identified, although other pathways may occur in certain species or tissues, or at certain developmental stages e.g., the pathway through galacturonate derived pectin from cell wall polysaccharides [2]. Several studies have identified that ascorbate concentrations in the cell are regulated mainly through the control of transcription of GDP-l-galactose phosphorylase (GGP), although GDP-d-mannose-3′,5′-epimerase (GME) and GDP-mannose pyrophosphorylase (GMP) may also contribute to a lesser extent (Figure 1). A feed-back regulation system at the translational level has been identified [29] and is mediated through a conserved upstream open reading frame (uORF), present in the 5′ untranslated region of the messenger RNA (5′UTR) of GGP. In Arabidopsis there are two functional *GGP* loci, referred to as *VTC2* and *VTC5*, with *VTC2* being the most highly expressed of the two in nearly all tissues [30]. In regard to the uORF feed-back system, an increase in AsA is sensed by an as yet unidentified mechanism, which then results in a reduction of the translation of the GGP enzyme protein. The predicted small uORF peptide (as yet undetected *in planta*) has features similar to that of ribosome inhibitors, so it is thought that translation initiation at the uORF results in stalled transcripts [29].

With regard to regulation of AsA biosynthesis, a number of regulators have been associated with affecting the transcription of various biosynthetic steps (Figure 1). These include an F-box type repressor known as *ascorbic acid mannose pathway regulator 1* (*AMR1*) [31], a positively regulating transcription factor HD-ZIP I (identified in tomato, gene named *SlHZ24*; *Solyc04g005800.2*) [32], and a positively regulating ethylene response factor subfamily b-3 transcription factor *AtERF98* (*At3g23230*) [33,34]. In addition, a number of genes were identified in a transcriptomics study in tomato of which three were verified by transient expression to positively up-regulate the expression of various genes involved in AsA biosynthesis [35]. They were *SlMYB91*: *Solyc09g010840.1*, a NAC (*JA2*) *Solyc12g013620.1*, and a zinc finger transcription factor *Solyc06g065440.1* (ZIF). Transient expression of all three generally increased early l-galactose pathway and galacturonate pathway genes in a similar manner while differentially activating different ascorbate peroxidases. *SlMYB91* and *JA2* also increased ascorbate oxidase expression which oxidizes AsA directly to dehydroascorbate (DHA) (2 L-ascorbic acid + O_2_ → 2DHA + H_2_O), and increased tissue-specific expression of specific isoforms of ascorbate oxidase in response to salinity/drought stresses has been observed in rice [36], suggesting that *SlMYB91* and *JA2* could be abiotic stress related. Indeed the Arabidopsis orthologue of *Solyc12g013620.1* (*JA2*) is *ANAC072* (*At4g27410*) which is a transcriptional activator in the abscisic acid (ABA) mediated dehydration response and is induced in response to desiccation, salt, cold, methyl jasmonate, and ROS (including H_2_O_2_) [37]. *ANAC019* (*At1g52890*) is also as close a match as *ANAC072* to *JA2*, and whose expression is induced by drought, salinity and ABA [38,39]. Both ANAC072 and ANAC019 activate transcription of the dehydration responsive *EARLY RESPONSIVE TO DEHYDRATION STRESS 1* gene (*ERD1*; *At5g51070*) [40]. The Arabidopsis orthologue of the zinc finger transcription factor *Solyc06g065440.1* is *STOP1* (*At1g34370*). STOP1 and its physiologically minor isoform STOP2 (*At5g22890*) regulate multiple genes critical for tolerance to aluminum [41,42]. Recently it was shown that root expressed ABI4 (*At2g40220*) binds the VTC2 promoter to suppress *VTC2* (*GGP*) transcription [43]. ABI4 is a transcription factor involved in ABA signal transduction and acts downstream of GUN1 (*At2g31400*) in retrograde signaling [44]. Most recently, a basic helix-loop-helix (bHLH) named *SlbHLH59* was identified in a genome-wide association study on 302 tomato accessions which positively regulates AsA in tomato fruits by directly binding to the promoters of the l-galactose pathway genes *phosphomannose mutase* (*PMM*) and *GDP-mannose pyrophosphorylase* (*GMP*) (with transcription of *GME* being stimulated in transient expression experiments) and for which the closest Arabidopsis protein is ‘unfertilized embryo sac 12’ (UNE12) [45]. *SlbHLH59* also possibly weakly represses GGP transcription (in one of two experiments) [45].

Various genes have also been identified that have protein interactions with GMP (*VTC1* in Arabidopsis; *At2g39770*). These include two genes: *CSN5B* and *CSN8* (*At1g71230* and *At4g14110*) which form separate subunits of the COP9-signalosome complex which promotes ubiquitin-dependent degradation of GMP through the 26S proteasome pathway [46,47]. Additionally, a tomato C2H2 Zinc Finger rapidly induced by NaCl treatment named *SlZF3* was identified which binds to CSN5B to inhibit CSN5B binding to GMP [48]. The closest Arabidopsis protein match to SlZF3 is ZAT12 (At5g59820.1) and this and related genes such as *ZAT10* (*At1g27730*) are associated with response to abiotic stress [49,50,51]. Two other proteins: *KONJAC1* (At1g74910) and *KONJAC2* (*At2g04650*), have been shown to stimulate recombinant GMP activity in vitro through protein–protein interaction. Curiously, although it is not required for GMP activity in vitro, the mutant *KONJAC1* plant line *kjc1-1* (SALK_044963) has GMP activity of only 10% wild-type levels, which resulted in a 60% reduction in AsA levels. The corresponding plant mutant line of *KONJAC2*, *kjc2-1* (SALK_023876), had ~80% wild-type GMP activity and the *kjc1-1 kjc2-1* double mutant had 8% wild-type GMP activity and exhibited severe dwarfism [52]. Finally a calmodulin-like protein named CML10 in the presence of Ca^2+^ was shown to have a small effect on PMM activity (*At2g45790.1*), suggesting that calcium status could also be modulating AsA metabolism [53].

Various abiotic inputs such as light and stresses such as salt, UV, ozone, metals, nitric oxide, temperature extremes and drought are also known to influence AsA metabolism [25,34,54,55,56,57,58,59,60,61,62,63,64,65,66,67,68,69,70]. Light is an elicitor of AsA metabolism and AsA undergoes diurnal change in leaves [30,47,69,71] although Laing et al. [72] did not observe diurnal changes in ascorbate in Arabidopsis, even though genes for GGP and GME showed strong diurnal trends. That study used mature plants, not seedlings and did report long-term responses to growth in response to differing photon flux densities, but not short-term changes. *GGP* shows a significant response to different light levels [72], due to light-responsive *cis*-elements present in its promoter [73]. Other light responsive *cis*-elements have also been identified in the promoters of *l-galactose-1-phosphate phosphatase* (*GPP*) and *GLDH* in rice [74]. The *vtc3* mutant is defective in its ability to elevate the AsA pool in response to light and heat, and VTC3’s unique dual function protein kinase: protein phosphatase activity suggests a role in mediating light and heat signals [75].

In its many interactions throughout the cell, AsA becomes oxidized to monodehydroascorbate (MDHA; also referred to as ascorbate free-radical) and if oxidized again becomes dehydroascorbic acid (DHA). If not re-reduced quickly, the pool of MDHA will react with itself (dis-proportionate) to form AsA and DHA. Likewise, if DHA is not re-reduced, it is broken down and lost, and so if not recycled, the AsA pool would eventually be lost. In living systems, an active recycling system is in place to maintain AsA homeostasis through reducing MDHA, as well as glutathione-dependent DHA reductase enzymes (MDHAR and DHARs, respectively). These genes exist as multigene families, and the various isoforms differ in their spatial, temporal and cellular organelle expression, and also respond to varying environmental inputs. In the case of DHA reduction performed by DHAR, the reducing power is donated by glutathione and so this process is coupled with the glutathione cycle and glutathione reductases [76].

In this study we elevated the *Arabidopsis thaliana* ‘Col0’ rosette AsA pool artificially, in the absence of direct abiotic and biotic factors through feeding AsA precursors l-galactose (l-gal) or l-galactono-1,4-lactone (l-galL), as well as AsA, with glucose (glc) used as control, over a period of 24 h. We then looked for any changes in gene expression of the genes discussed previously by quantitative PCR. Network analysis and gene ontologies suggested links with phytohormones such as abscisic acid and jasmonates so we measured a subset of phytohormones as well. From this we present evidence of how gene transcripts of known and deduced AsA related genes change in response to an increase in AsA concentration in the absence of external abiotic stimuli (e.g., a change in light irradiance) to obtain greater insights into the regulation of AsA metabolism and how it is involved in abiotic stress tolerance.

## 2. Results

### 2.1. AsA Precursor Feeding

**Figure 2:**
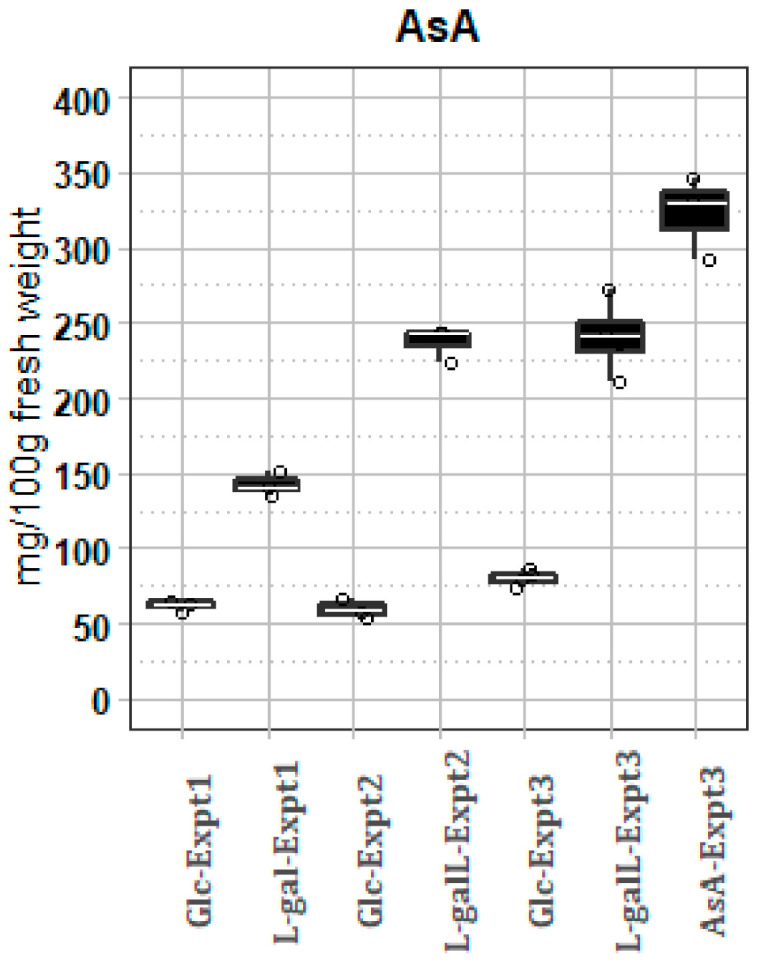
Increase in AsA content after feeding either 25 mM l-gal, l-galL, or AsA, with 25 mM glc used as control. Error bars are standard deviations (n = 3 for experiments 1 and 2 and n = 4 for experiment 3). Plant ages: experiment 1 = 21 days post sowing plants; experiment 2 = 38 days post sowing plants; experiment 3 = 27 days post sowing plants.

Arabidopsis plants were sprayed with a perfume sprayer to wetness (fine bubbles on leaf) four times over a 24 h period with 25 mM solutions of l-galactose pathway intermediates l-gal (21 days post sowing plants; experiment 1) or l-galL (38 days post sowing plants; experiment 2 and 3), as well as 25 mM AsA (27 days post sowing plants experiment 3). Glucose (25mM) was applied as a control and these plants were used as the baseline controls for comparisons. Plant rosettes were harvested 4 h after the final spray, which corresponded to 28 h of exposure to substrate. Spraying with l-gal, l-galL, and AsA consistently caused large increases in rosette total AsA, averaging 2.3-fold, 3.9-fold, and 4.2-fold, respectively, with low inter-plant variation (Figure 2). The low inter-plant variation suggests that the substrates application rates were saturating. Control glc treatment AsA levels were very similar for experiments 1 and 2, but experiment 3 glc control was marginally higher (1.29- and 1.34-fold) than that of experiments 1 and 2 glc controls, respectively (*p* < 0.01 for both; Student’s *t*-test). This may reflect seasonal effects. Note that feeding AsA would be physiologically different from feeding the two precursors. Firstly, the precursors would result in AsA being synthesized in the mitochondria, the location of GalLDH and it is unlikely any AsA was made extracellularly. AsA would accumulate extracellularly where it may be degraded [7] and activate redox reactions [77].

### 2.2. Gene Expression Results

For most of the genes, measured gene expression varied very little but for those that did, expression ratios of treatments over controls varied in the order of 0.1- to 20.7-fold. The differentially expressed genes varied by treatment and the results are broken down by area of metabolism in the following sections. The list of genes including primer sequences are listed in supplementary data (Table S1).

### 2.3. l-Galactose Pathway Gene Expression, Including Pre-Pathway Genes as Well as Alternate Galacturonate Pathway

**Figure 3:**
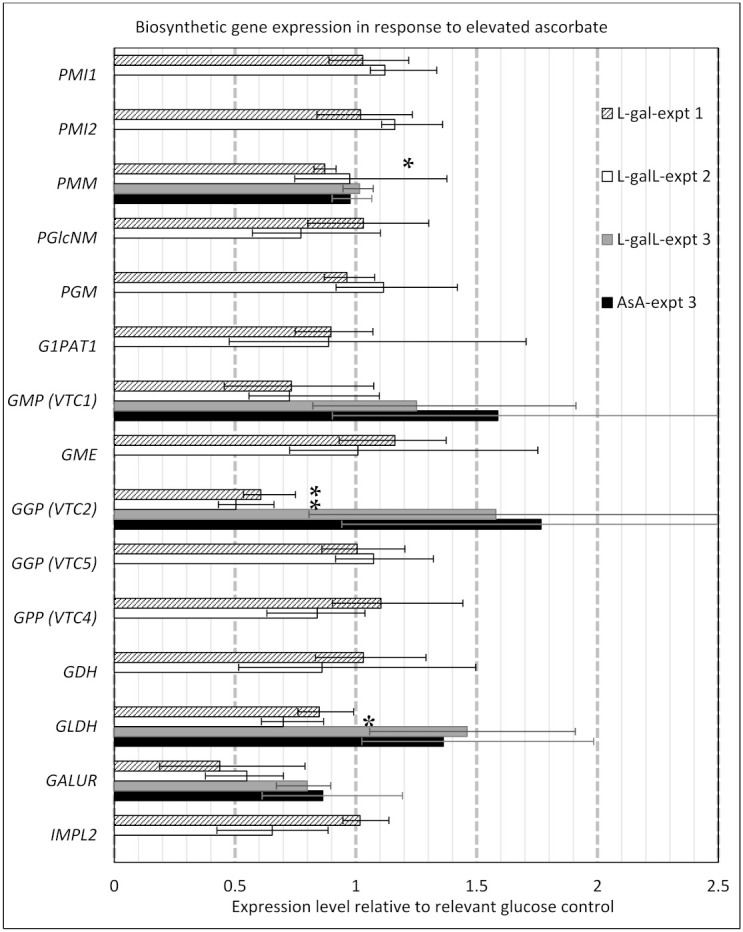
Response of biosynthetic gene expression to elevated AsA. Error bars represent standard error of the mean. Significant differences to the respective control are indicated with an asterisk (*p* < 0.05). *PMI1* (*At3g02570.1*) and *PMI2* (*At1g67070.1*): mannose-6-phosphate isomerase; *PMM* (*At2g45790.1*): phosphomannomutase; *PGlcNM* (*At5g17530.1*): phosphoglucosamine mutase; *PGM* (*At1g70820.1*): phosphoglucomutase; *G1PAT1* (*At4g30570.1*): Glucose-1-phosphate adenylyltransferase/GDP-mannose pyrophosphorylase; *GMP* (*VTC1*) (*At2g39770.1*): GDP-mannose pyrophosphorylase; *GME* (*At5g28840.1*): GDP-d-mannose 3′,5′-epimerase; *GGP* (*VTC2*) (*At4g26850.1*) and *GGP* (*VTC5*) (*At5g55120.1*): GDP-l-galactose phosphorylase; *GPP* (*VTC4*) (*At3g02870.1*): l-galactose-1-phosphate phosphatase; *GDH* (*At4g33670.1*): l-galactose dehydrogenase; *GLDH* (*At3g47930.1*): l-Galactono-1,4-lactone dehydrogenase; *GalUR* (*At1g59960.1*): d-galacturonate reductase; *IMPL2* (*At4g39120.1*): myo-inositol monophosphatase.

Increasing AsA elicited few effects on the expression of the genes involved in biosynthesis pathways. Only three genes were shown to have statistically different gene expression and this varied by type of AsA precursor that was applied (Figure 3). The biosynthetic genes for which transcripts changed in response to elevated AsA included: phosphomannose mutase (*PMM-At2g45790.1*) barely changed (but judged statistically significant) at only 0.87-fold that of the control in l-gal treated plants only; and l-Galactono-1,4-lactone dehydrogenase (*GLDH*-*At3g47930.1*): 0.69-fold control in l-galL treated plants only. *GGP* was affected by both l-gal and L-galL treatments in experiments 1 and 2, and reduced to 0.61 and 0.50-fold respectively, but was not significantly different in experiment 3. Transcripts for the physiologically minor isoform the *GGP* (*VTC5*; *At5g55120*) were unchanged.

### 2.4. Ascorbate Oxidase and AsA Recycling Genes

**Figure 4:**
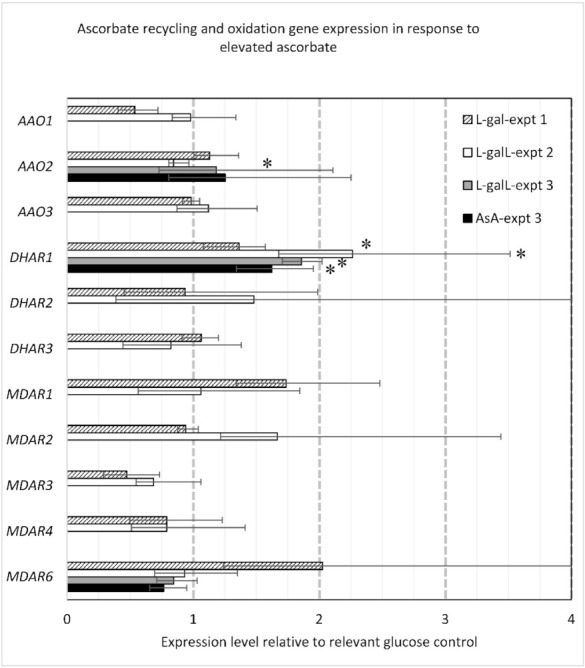
Response of AsA recycling gene expression to elevated AsA. Error bars represent standard error of the mean. Significant differences to the respective control are indicated with an asterisk (*p* < 0.05). *MDAR6* (*At1g63940.1*), *MDAR3* (*At3g09940.1*), *MDAR4* (*At3g27820.1*), *MDAR1* (*At3g52880.1*), *MDAR2* (*At5g03630.1*); *DHAR1* (*At1g19570.1*), *DHAR2* (*At1g75270.1*), *DHAR3* (*At5g16710.1*); *AAO1* (*At4g39830*), *AAO2* (*At5g21100*), *AAO3* (*At5g21105*).

The expression of known *Arabidopsis* genes in the AsA recycling pathway as well as three annotated Ascorbate oxidases was measured. Of the recycling genes, only *dehydroascorbate reductase 1* (*DHAR1*; *At1g19570.1*) was differentially expressed, up 1.36- to 2.26-fold in all AsA elevated treatments (Figure 4). Typically *DHAR1* is the dominantly expressed isoform in rosette tissues, ranging from 2- to 4-fold higher than *DHAR3* and from 18- to 38-fold higher than *DHAR2* (data from [72]). Four of the five MDHARs appeared to have altered expression but these were not deemed significant due to high variability. The interpretation could be that the response to elevated AsA is short lived or that gene expression levels were still in the process of normalizing to a different expression level or else their expression is just naturally highly variable. Typically in rosettes *MDHAR*s *4*, *1*, and *2* are the most highly expressed, ranging on average from 5- to 31-fold higher than *MDHAR*s *6* and *3* [72].

For the ascorbate oxidases, *AAO2* expression was reduced to 0.84-fold compared with the control but only in L-galL treated plants (experiment 1; *p* < 0.05) (Figure 4). *AAO1* also appeared down in L-gal treated plants at 0.54-fold control but this was not significant, while *AAO3* expression was unchanged. Typically in rosette tissue, *AA02* is the most highly expressed isoform, ranging from 1.2- to 6.1-fold higher expression than AA03 and from 3.3- to 54.2-fold higher expression than *AA01* (data from [72]).

### 2.5. Post Translational Modulators

There was no change in expression for genes known to affect PMM and GMP protein activity such as *CML10* and *KONJAC1* and *2*. Expression of the *VTC3* regulatory gene was unchanged as well. No change in expression of *CSN5B* and *CSN8* (components of the COP9 complex promoting GMP degradation) suggests that if there is a feedback response through reduction of GMP enzyme levels, it had not yet emerged at this time of measurement. *GMP* (*VTC1*) transcripts were potentially reduced (although not significantly). Expression data for these post translational modulators are not presented but ranged from 0.48-fold control for *KONJAC2* (not significant) and from 0.85- to 1.15-fold respective controls for the remainder.

### 2.6. Expression of Known Regulatory Genes

The closest *Arabidopsis thaliana* orthologues to the tomato regulatory genes described in the introduction were identified by BLASTP [78]. For most genes it was clear what the closest match was, however, this was not the case for others such as *SlZF3* and *SlZH24.* The closest Arabidopsis protein match to SlZF3 was ZAT12 (*At5g59820*), a zinc finger protein involved in high light and cold acclimation [49,79,80], which shared 41.5% amino acid identity, but three other zinc finger genes were also close and shared 36.9–38.7% identity (ZAT11*-* At2g37430; At3g46080; and At2g28710). The latter three were not measured because they had very low or no expression in rosettes (‘ePlant’ browser and data from [72]). Two NAC genes were close protein matches to tomato JA2 (*Solyc12g013620.*1): ANAC019 and ANAC072 (56–57% protein identity), so both were measured. Determining the exact Arabidopsis orthologue for *SlZH24* (*Solyc04g005800.2*) was the most problematic because it most closely matched with the large homeodomain leucine zipper class I (HD-Zip I) protein family (23–28% amino acid identity; Figure S1). Based on this, *ATHB1*, *ATHB6* and *ATHB12* were chosen for the initial screen. *ATHB1* was of additional interest because it harbors an upstream open-reading frame in its 5-UTR region and also has a natural antisense gene (*At3G01035*) whose transcript overlaps with *ATHB1* mRNA, so this was measured as well. ABI4 has been linked to repressing *GGP* expression but no expression was detected in vegetative rosettes, and this is consistent with publicly available expression data [72]. *ABI4* transcripts are mainly expressed in 24 h imbibed seed and elongating portions of roots, and tend to be more expressed in the dark (expression data source: ePlant browser); our samples were harvested at midday. The upstream indirect repressor of *ABI4*, *EIN3*, was also measured but expression was unchanged, as was the expression of the potential positive transcriptional regulator *UNE12*, the closest Arabidopsis match to tomato *SlbHLH59*.

**Figure 5:**
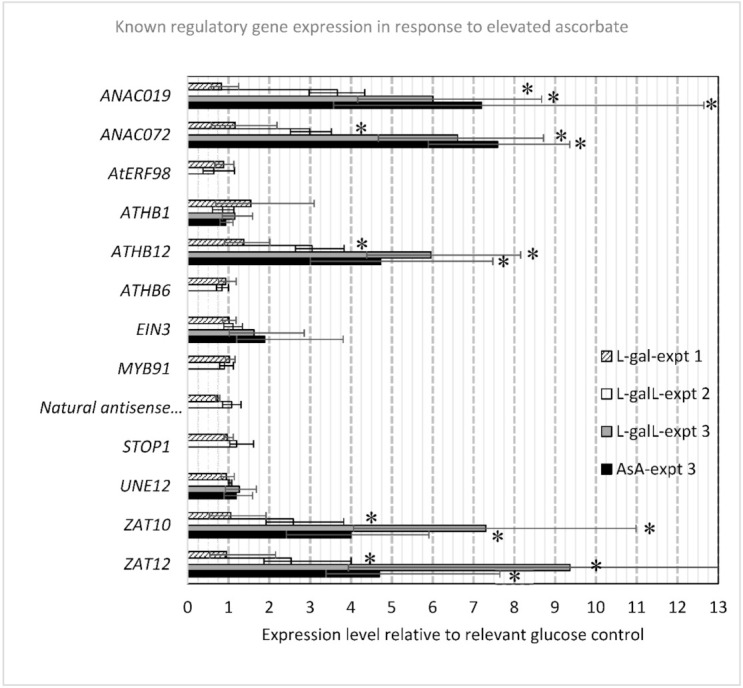
Response of known regulatory genes or close orthologues thereof to elevated AsA. Error bars represent standard error of the mean. Significant differences to respective control are indicated with asterisk (*p* < 0.05). *ANAC019* (*At1g52890*); *ANAC072* (*At4g27410*); *AtERF98* (*At3g23230*), *ATHB1* (*AT3G01470*); *ATHB12* (*AT3G61890*); *ATHB6* (*AT2G22430*); *EIN3* (*AT3G20770*); *MYB91* (*At2g37630*)- closest match to *SlMYB91*; natural antisense to *ATHB1* (*At3g01035*); *STOP1* (*At1g34370*); *UNE12* (*AT4G02590*); tomato *SlZF3* orthologues *ZAT12* (*At5g59820*) and *ZAT10* (*At1g27730*).

Expression of all the regulatory genes discussed previously was measured and five of these: *AtHB12* (up 3.05- to 5.96-fold), *ANAC019* (up 3.67- to 7.2-fold), *ANAC072* (up 2.99- to 7.59-fold), *ZAT12* (up 2.54- to 9.36-fold) and *ZAT10* (up 2.59- to 7.3fold) had statistically different gene expression, but only for L-galL and AsA treatments (Figure 5). *AtHB1*, a positive transcriptional regulator associated with response to desiccation, was not statistically different. Expression of *STOP2* (physiologically minor isoform of *STOP1*) and *AMR1* was low/not detected so data for these are not presented.

### 2.7. Exploring Regulatory Network

**Figure 6:**
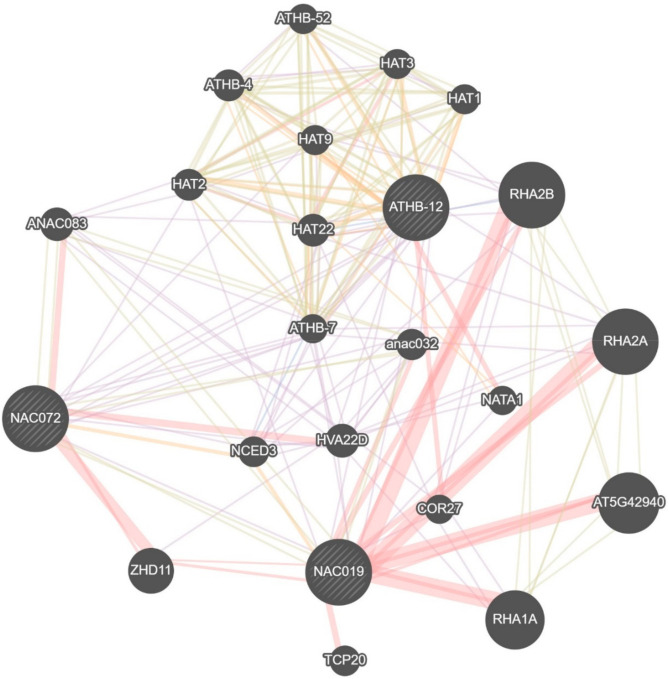
Gene interactor network of *ATHB12*, *ANAC019* and *ANAC072* as a query set. Red = physical interactions; orange = predicted; lilac = co-expressed; green = genetic interactions; blue = co-localization (see genemania.org). Gene functions: *RHA(1A/2A/B)* and *At5g42940*: involved in protein ubiquitination/degradation processes regulating ABA signaling and mediating response to abiotic stress; *COR27*: role in integrating circadian clock and cold response; NATA: ornithine N-delta-acetyltransferase-producing defense compound; HVA22D: ABA- and stress-inducible gene; ZHD11 (ATHB29): zinc finger homeodomain transcriptional factor which binds to *ERD1* promoter (*Early Responsive to Dehydration stress 1*); *NCED3* (*At3g14440*): 9-cis-epoxycarotenoid dioxygenase, a key enzyme in the biosynthesis of ABA, and regulated in response to drought and salinity; *ATHB-4/7/52* HD-Zip I transcription factors: shade avoidance/ABA mediated drought response/mediates crosstalk between ethylene and auxin by transcriptionally modulating *PIN2*, *WAG1*, and *WAG2*; *ANAC032/083*: modulates JA/SA signaling/negatively regulates xylem vessel formation; *HAT1/2/3/9/22*–HD-Zip II regulatory proteins/transcription factors: negatively regulates ABA synthesis and signaling in drought response/induced by auxin, but not by other phytohormones, regulates auxin-mediated morphogenesis/controls leaf development/dehydration stress memory/ABA and stress-inducible.

Seeing that *ATHB12*, *ANAC019* and *ANAC072* transcription had been stimulated by elevated AsA, we looked for other potentially affected genes by querying the ‘GeneMANIA’ prediction server [81] in order to identify interacting partners. This revealed a shared co-network of genes of known interactors for *ATHB12*, *ANAC019* and *ANAC072* (Figure 6). Based upon abiotic stress related gene ontology annotation, the expression of a number of these were measured and included *NCED3*, *ATHB54*, *ATHB52*, *ATHB7*, *ATBH16*, *HAT2*, *ZHD11*, *ABI5* (for *ANAC019* and *ANAC072* predicted interaction network, not shown in Figure 6), and *ABF3* (only for *ANAC072* network, not shown in Figure 6). *TCP13*, an upstream negative regulator of *ATHB12* was also included as was *Early Responsive to Dehydration stress 1* (*ERD1*; *At5g51070*) which is regulated by ZHD11 [82].

**Figure 7:**
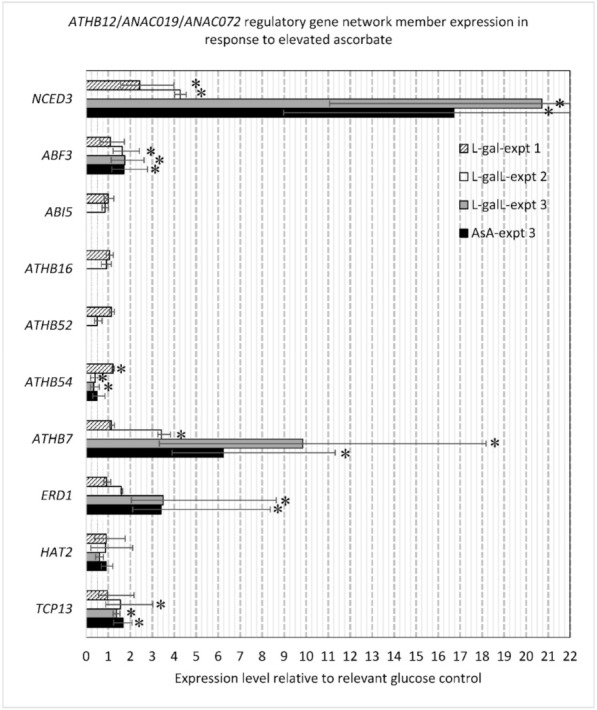
Response of selected genes in putative ATHB12/ANAC019/ANAC072 gene network to elevated AsA. Error bars represent standard errors. Significant differences to the respective control are indicated with an asterisk (*p* < 0.05). Gene functions: *NCED3* (*At3g14440.1*): 9-*cis*-epoxycarotenoid dioxygenase, a key enzyme in the biosynthesis of ABA; *ABF3* (*At4g34000.1*): ABA-responsive element/transcription factor expressed in response to stress and abscisic acid; *ABI5* (*At2g36270.1*): bZIP involved in ABA signaling; *ATHB16* (*At4g40060.1*): transcription factor involved in photoperiodism and transition to flowering; *ATHB52* (*At5g53980.1*): transcription factor mediating crosstalk between ethylene and auxin; *ATHB54* (*At1g27045.1*): transcription factor; *ATHB7* (*At2g46680.1*): ABA regulated and may act in a signal transduction pathway mediating drought response; *ERD1* (*At5g51070.1*): *Early Responsive to Dehydration stress 1*, ATP-dependent Clp protease regulatory subunit (mRNA is cell-to-cell mobile); *HAT2* (*At5g47370.1*): HB-ZIP induced by auxin; *TCP13* (*At3g02150.2*): upstream regulator of *ATHB12*.

Of the ten genes measured from the gene interactor network, six displayed altered transcript levels in response to elevated AsA mainly through l-galL and AsA treatments (Figure 7). The clearest response was observed for *NCED3* (up 2.43- to 20.72-fold; l-gal, l-galL and AsA) and *ATHB7* (up 3.42- to 9.84-fold; l-galL and AsA). *ERD1* expression was only significant in experiment 3 for l-galL and AsA treatments (up 3.40- to 3.50-fold). *ATHB54* was up slightly for l-gal treatment (1.22-fold; *p* < 0.5) but down 0.34- to 0.49-fold control for l-galL and AsA treatments. Transcripts for the ABA-responsive element-binding protein *ABF3* was up 1.64- to 1.75-fold for l-galL and AsA treatments, and *TCP13*, the upstream negative regulator of *ATHB12* was up 1.38- to 1.68-fold compared to controls. *ZHD11* expression was not detected and this is not unexpected because mainly expressed in roots and these tissues were not measured.

### 2.8. Changes in Phytohormone Content in Response to Elevated AsA Content

**Figure 8:**
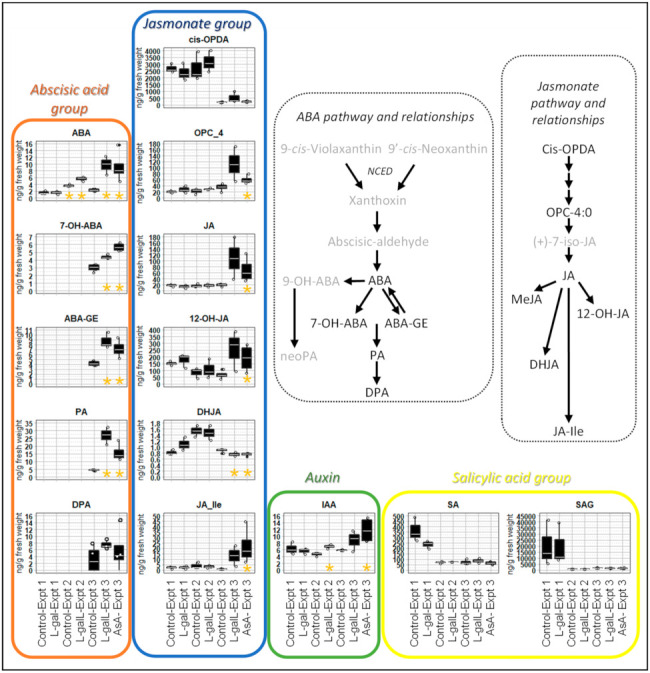
Boxplots highlighting changes in phytohormone content (ng/g fresh weight) in response to elevating AsA levels. Gold asterisks denote statistically different treatments to their respective glc controls (*p* < 0.05; Student’s *t*-test). Inset panels for ABA and jasmonate show their biosynthetic pathways in order to demonstrate how the individual compounds relate to each other with compounds for which there is data being in black font while compounds not measured are in grey font. Note that biosynthetic pathways are not definitively depicted, and multiple consecutive arrows denote multiple consecutive biosynthetic steps. Step catalyzed by 9-cis-epoxycarotenoid dioxygenase (*NCED*) is shown in italics.

Gene ontology metadata for many of the aforementioned genes showed links to roles in jasmonate and ABA phytohormone signaling processes. We therefore measured subsets of jasmonate, ABA, auxin, and salicylic acid (SA) classes of compounds for the three separate experiments, adding in the measurement of additional compounds 7′-hydroxy ABA (7-OH-ABA), ABA-D-glucopyranosyl ester (ABA-GE), phaseic acid (PA) and dihydro-phaseic acid (DPA) for experiment 3 (Figure 8; Table S2). l-galL and AsA treatments had relatively large increases in certain jasmonates (particularly bioactive Ja-Ile) in experiment 3 at 14- and 24-fold respectively, but l-galL treatment in experiment 2 showed no change. Note that Ja-Ile in l-galL treatment in experiment 3 was 14-fold higher than the control but was not judged statistically significant (*p* = 0.07) (data in Table S2). Methyl-jasmonate (MeJA) was measured but not detected in any of the samples, glc controls included. There was a slight decrease (0.85-fold control; *p* < 0.05) in 9,10-dihydro-jasmonic acid (DHJA) in experiment 3 only (l-galL and AsA treatments). DHJA is associated with growth inhibition, induction of senescence and in some cases induction of alkaloid and nicotine synthesis [83,84].

Increases in ABA were most consistent, showing no change for l-gal treatment, but 1.54- to 4.26-fold increases for l-galL and AsA treatments. The ABA catabolic derivative (but still bioactive) phaseic acid (PA) was elevated 6.05- and 3.55-fold in l-galL and AsA treatments, respectively. The inactive ABA catabolites 7-OH-ABA and ABA-GE were both increased by l-galL and AsA treatments (in range of 1.5- to 2-fold). Auxin in the form of indole-3-acetic acid (IAA) increased 1.5- to 2-fold for L-galL (only statistically different in experiment 2) and AsA treatments. SA and SA O-β-glucoside (SAG; inactive storage form of SA) were unaffected by increases in AsA levels.

## 3. Discussion

In this study we confirm that AsA content in Arabidopsis can be markedly increased by foliar sprays of the l-galactose AsA pathway precursors L-gal and L-galL, as well as AsA itself. Applications over a 24 h period caused large 2.3- to 4.3-fold increases in AsA content over respective glc controls. L-galL was more effective than L-gal which may be expected as it is the last step before ascorbate. However, this is complicated by differing ages of the sets of plants at time of treatment: 21 days (experiment 1; 2.3-fold increase) for L-gal, 38 days (experiment 2; 3.9-fold increase) and 27 days (experiment 3; 3-fold increase), with each having the same age respective glc controls; therefore, there could be a confounding effect of vegetative maturity on the relative effectiveness of the particular substrate, the trend of greater increases in older rosettes for L-galL lends support to this. An additional factor could be conversion rate; when the absolute expression levels of the four references, as well as *GDH* and *GLDH* gene transcripts were compared between experiments 1 and 2, there was no clear evidence of a difference in overall transcript number (data not presented). This conclusion takes into account the ratio between reference gene and unchanged transcripts, which were very similar. Under the assumption that the reference gene transcripts were stable between the different ages of vegetative rosette, it is possible that there is indeed a difference in conversion efficiency between L-gal and L-galL. Furthermore, as the end-point product AsA was also applied and showed the highest increase, it also lends support to a difference in conversion time. The vegetative rosettes were harvested at the same time period after treatment was finished, so the time available for conversion to AsA was the same, and thus time for conversion could be a cause of the difference in AsA increases observed.

Putting this aside, the relevant point for this study is that AsA contents were significantly altered in a relatively short period of time without any change in environmental inputs. It was thought that this approach would have fewer pleiotropic effects compared to other methods, such as a high light treatment, and thus direct feedback events could be observed. Most of the differential gene expression changes were for L-galL and AsA treatments which elevated AsA content more than L-gal, suggesting there could be a concentration threshold that must be surpassed before many of the observed effects on gene expression are enacted.

For biosynthetic gene expression there was generally little change in response to elevated AsA content. Transcripts for *GGP* (*VTC2; At4g26850.1*) which is an important control point in AsA biosynthesis [85], were reduced in experiments 1 and 2 to approximately 40–50% that of the glc controls for L-gal and L-galL treatments, but no significant change was observed in experiment 3. None of the transcription factors known to affect *GGP* transcription that was tested had statistically different expression from the glucose control. Both AMR1 and ABI4 have been shown to transcriptionally repress *GGP* [31,43], but we did not detect transcripts for either gene in rosette tissue, which is not unexpected as *AMR1* is either not expressed or expressed extremely lowly [72,86] and its expression is down-regulated in light [31] (samples taken around midday), and *ABI4* is mainly expressed in root and imbibed seed tissues, thus it appears that regulation of *GGP* differs between roots and leaves (rosette in this case).

Other biosynthetic genes which were changed were *PMM* and *GLDH*, but this did not occur in all treatments. Therefore, there appears to be little or no biosynthetic gene transcriptional feedback response to elevated AsA.

AsA recycling is a major component of AsA metabolism and is responsive to AsA redox status, and biotic and abiotic stress. Since the AsA elevated plants were not in a stressed condition, artificial AsA elevation was not expected to impact on AsA recycling gene expression, but unexpectedly *DHAR1* (*At1g19570.1*) was up 1.4- to 2.3-fold in response to the three different treatments. It is unclear why this occurred, but as there was a large increase in the pool of total ascorbate this could be eliciting an increased recycling response.

The response of *DHAR1* expression could instead be due to downstream effects of elevated AsA which induced a change in phytohormones, which in turn likely activated the expression of transcription factor genes associated with abiotic stresses. There are numerous reports linking increased AsA (as well as AsA recycling) with increased tolerance to abiotic stresses, drought and salinity in particular [33,54,87,88,89,90,91,92,93,94,95,96,97]. Abiotic stress leads to a range of alterations in metabolism, altered phytohormone balances, ROS imbalances, and formation of stress metabolites are perceived and integrated by triggering signal transduction pathways which further impact hormone and ROS levels. All these changes lead to a wide range of responses which result in acclimation to the original stress [98].

The exact mechanism of how increased AsA causes increases abiotic stress tolerance, apart from its dogmatic role in controlling ROS and links with hormone metabolism, remains unclear. Here we found that an increase in AsA exclusive of other known AsA inductive treatments such as high light, increased the concentration of ABA, PA, certain jasmonate phytohormones, and a small increase in auxin (IAA). Ethylene, gibberellins, brassinosteroids and strigolactones were not measured so there is little to comment on. However, out of all the genes measured, only *AtHB52* shows responsiveness to 1-aminocyclopropane-1-carboxylic acid (ethylene precursor) treatment [99] and its expression was slightly reduced, suggesting ethylene levels were not stimulated by elevating AsA.

ABA and to a lesser degree IAA were increased in a most consistent fashion while changes in jasmonates such as precursors OPC-4 and JA, catabolites 12-OH-JA and DHJA, and bioactive JA-Ile were inconsistent between experiments. The increase in ABA is most likely due to increased expression of transcripts for 9-cis-epoxycarotenoid dioxygenase (*NCED3*), a key enzyme in the biosynthesis of ABA [100], with *NCED3* being the main stress-induced NCED in leaves [101]. *NCED3* was identified in the gene interactor network using a query set of *ATHB12*, *ANAC019* and *ANAC072* (Figure 6). Expression of the other *NCED* genes was not measured. It is known that both ascorbate and glutathione are accumulated under progressive water stress and that this is mediated by an early accumulation in ABA and increase in ROS [102]. It is also known that ascorbate integrates the antagonistic modulation of ethylene and ABA [43]. Here we found that this crosstalk is bi-directional. Most of the upregulated genes are upregulated by ABA treatment alone so the key AsA responsive gene(s) are yet to be identified. However, transcripts for *NCED3* were highly upregulated and so this could be a directly AsA responsive candidate even though it is also upregulated by ABA alone (~5-fold) (see supplementary data in [103]).

**Figure 9:**
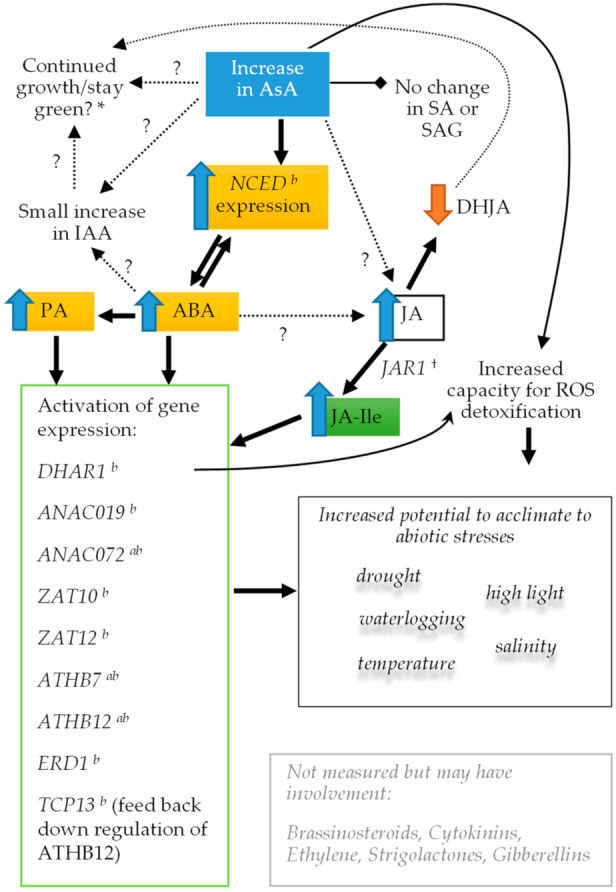
Potential scheme for how elevated AsA (in absence of other stimuli) causes changes in signalling and confers abiotic stress tolerance/acclimation. Blue or orange filled arrows denote increase or decrease (resp.) in the associated compound or gene expression. Gene expression was cross referenced to supplementary data from Weng et al. 2016 [102] to identify whether ABA, PA or DPA treatments alone can elicit gene expression and this is indicated by ^a^ for PA or ^b^ for ABA (DPA did not change expression of any genes discussed here so not shown). DHJA promotes senescence [104]. * Increased AsA is associated with increased chlorophyll a/b. ^†^ *JAR1* (jasmonate-amido synthetase; At2g46370) is not induced by ABA, PA or DPA. Possible weak induction by IAA [98,102].

Therefore, it seems that stimulation of phytohormone production, such as ABA and JA-Ile, and subsequent downstream signal transduction outcomes likely explain the increased tolerance to abiotic stresses (drought, salt and cold) observed when AsA contents are increased by transgenic methods (Figure 9). ABA, PA, jasmonates and brassinosteroids are key phytohormones mediating responses to abiotic stresses. In particular, we found PA increased 3.5- to 6-fold. PA is an oxidative catabolite of ABA which selectively activates a subset of ABA receptor family members which fine-tunes physiology, environmental adaptation and development [103].

Thus, in addition to increased ROS detoxification though its intrinsic anti-oxidant properties, higher AsA confers stress tolerance through retrograde signaling to activate a range of stress response regulator genes such as *ANAC019* (drought, high salt), *ANAC072* (response to desiccation), *ATHB7* (drought response), *ATHB12* (drought response), *ABF3* (response to ABA), *ZAT10* (salt and photo-oxidative stress) and *ZAT12* (high light and cold acclimation), and that this response could be dependent on surpassing a yet to be determined concentration threshold. The differential ABA response is also interesting, for example, activation of *ABF3* but not *ABI5* (which is highly induced by ABA but not PA or DPA [103]; Figure 7), and suggests a nuanced role for the AsA retrograde signal.

## 4. Materials and Methods

‘Col0’ Arabidopsis plants were sown directly in soil contained within 10 cm square pots and grown in a green house. They were grown in and kept well-watered until the experimental treatment was imposed. Multiple plants were grown in each pot, and each of three separate pots were regarded as a replicate. The compounds were applied by using a 1 mL hand-operated pump perfume sprayer that generated a fine mist. Plants were sprayed until the leaves were covered in small droplets (about 1.3 mL of solution over the three pots per compound), which dried rapidly and left no observable residue. Each pot was harvested separately after 28 h and frozen in liquid nitrogen (whole rosettes taken and pooled for each pot). AsA was measured by HPLC as described previously [105]. Experiment 1 (l-gal and glc control treatments) was applied to 21 days post sowing plants, experiment 2 (l-galL and glc control treatments) was applied to 38 days post sowing plants, and experiment 3 (l-galL, AsA and glc control treatments) was applied to 27 days post sowing plants.

Total RNA was extracted from each biological replicate using a Spectrum™ Plant Total RNA Kit (Sigma-Aldrich Co, St. Louis, MO, USA), and inputted material amounts ranged between 50 to 100 mg frozen powdered tissue. Total RNA quality and quantity was evaluated by capillary electrophoresis with RNA StdSens analysis kit on an Experion™ instrument (Bio-Rad Laboratories, Inc., Hercules, CA, USA). All RNA samples were in the RNA Quality Indicator (RQI) ‘Acceptable quality’ bracket (7 to 10) and RQIs ranged from 8.1 to 9.7 (concentrations ranged from 160 to 735 ng/µL). PolyA+ mRNA was then purified from 10 µg total RNA input using 100 µL magnetic Oligo(dT)_25_ beads (Dynabeads mRNA Direct Kit, Thermofisher Scientific, Waltham, MA, USA), eluting in 23 µL. The purified polyA+ mRNA was quantified by capillary electrophoresis as before and 25 ng was used as a template for complementary DNA (cDNA) synthesis in a 10 µL total volume reaction using an iScript™ cDNA Synthesis Kit (Bio-Rad Laboratories, Inc.) using the following thermocycling program in a Perkin Elmer GeneAmp 9700 PCR Thermo Cycler (Perkin Elmer Inc., Waltham, MA, USA): 25 °C for 5 min, then 46 °C for 30 min, then 95 °C 1 min followed by cooling on ice. Upon completion the cDNA was diluted 10 times by adding 90 µL 10 mM Tris, 0.05 mM EDTA (pH8) buffer. One µL was used as template in a 15 µL total volume quantitative PCR reaction using PerfeCTa SYBR Green FastMix (2×) (Quantabio, Beverly, MA, USA) and 200 µM primers (custom synthesis by Macrogen Inc., South Korea). Each sample had three qPCR reactions with each representing one biological replicate consisting of separate RNA extraction, polyA+ mRNA purification and cDNA synthesis. In summary, the experiment consisted of two sets of two treatments of three separate biological replicates that yielded 12 samples in total.

Reactions were performed in a MIC qPCR Cycler (Bio Molecular Systems, Upper Coomera, Australia) with the following program: 95 °C 30 s; then 40 cycles of 95 °C 5 s, 60 °C 15 s, 72 °C 10 s (with data collection at end of each 72 °C step); followed by melt curve step of 72 to 95 °C at 0.3 °C/s, with continuous data collection. Some primer pairs had a higher annealing temperature of 63 °C if initial amplification was unsatisfactory. Poorly performing primer pairs were replaced with different primers but this was rare. Four genes were used as reference genes (described in [106]): *At1g13320.1* (encoding the 65 kDa regulatory subunit of protein phosphatase 2A (PP2AA3), *At1g59830.1* (one of the isoforms of the catalytic subunit of protein phosphatase 2A; PP2A-1); *At4g33380.1* (dimethylallyl, adenosine tRNA methylthiotransferase; DMATMT) and *At2g28390.1* (MON1). Primer sequences were mainly sourced from the ‘AtRTPrimer’ resource ([107]; http://atrtprimer.kaist.ac.kr/ accessed date 21 February 2021) using default conditions and a target amplicon size of 100–150 bp. Preference was given to primer pairs located in exons flanking an intron and where there was a large size difference between spliced mRNA CDS and genomic DNA amplification. In rare cases where no results were found the upper amplicon size limit was increased to 200 bp and if no suitable primers were returned after that then primers were designed using the Primer3 plugin [108] (100–150 amplicon size target; min Tm = 58, max Tm = 62, Optimum= 60) in Geneious^®^ 10.0.9 software (Biomatters Ltd., Auckland, New Zealand) or else using Primer-BLAST and NCBI [109]). All primer sequences are listed in Table S1.

Reactions were evaluated on their melt curve and non-amplification in no-template control samples. For some genes where there were failures, or what appeared to be outlier results in terms of divergent Cq values, additional repeat reactions were performed. In most cases the original outliers were included with the additional repeats in the final analysis. Purified amplicon of a number of genes (*VTC2*, *ANAC072*, *AAO1*, *AAO2*, and *AAO3*) were sequenced by Macrogen Inc. (Seoul, South Korea) to ensure correct target amplification and it was correct in all cases. Relative quantification was performed using the MIC software with the appropriate glucose treatment designated as the control which had an expression value of ‘1′ (glc1 used for l-gal and glc2 used for l-galL). Expression values of the gene of interest returned as a ratio of control sample expression. Data for all four reference genes were used for all analyses. The MIC software analyzes the expression of each gene incorporating normalization to the geometric mean of reference gene expression using geNorm [110]. In addition, Cqs are calculated and the efficiency of amplification data from each reaction is calculated using LinRegPCR [111]. Data output includes mean expression ratio [112], standard errors, 95% confidence intervals and an estimate of significance by REST analysis [113] (significance threshold set at *p* < 0.05).

### Phytohormones

Materials: Formic acid (Riedel-de Haën) was purchased from Sigma Aldrich (Auckland, New Zealand). Optima LC/MS grade acetonitrile and trifluoroacetic acid (TFA) were purchased from Thermo Fisher Scientific (Auckland, New Zealand). Water was of Milli-Q grade. Jasmonic acid (JA), salicylic acid (SA), abscisic acid (ABA) and methyl jasmonate (MeJA) were purchased from Sigma Aldrich (Auckland, New Zealand). Jasmonoyl-isoleucine (JA-Ile), 12-hydroxyjasmonic acid (12-OH JA), 9,10-dihydrojasmonic acid (DHJA), cis-(+)-12-oxo-phytodienoic acid (cis-OPDA), (+/−)-4-(3-oxo-2-(pent-2-enyl)cyclopentyl) butanoic acid (OPC4) and indole-3-acetic acid (IAA) were purchased from Olchemim Ltd. (Olomouc, Czech Republic). Phaseic acid (PA), dihydrophaseic acid (DPA), 7′-hydroxyabscisic acid (7′-OH-ABA) and abscisic acid D-glucopyranosyl ester (ABA-GE) were purchased from the National Research Council Canada (Saskatoon, Saskatchewan, Canada). [^2^H_5_] JA, [^2^H_4_] SA and [^2^H_5_] MeJA were purchased from CDN Isotopes (Pointe-Claire, Quebec, Canada), [^2^H_6_] ABA was purchased from Toronto Research Chemicals (Toronto, ONT, Canada) and [^13^C_6_] IAA was purchased from Cambridge Isotopes (Andover, MA, USA). Salicylic acid O-β-glycoside (SAG) was synthesized following published methodology ([114], and was >99% pure by ^1^H- and ^13^C-NMR. [^2^H_10_] JA-Ile was synthesized using a modification to published methodology by utilizing [^2^H_10_] L-isoleucine in place of isoleucine [115], and was >99% pure by liquid chromatography mass spectrometry (LC-MS). [^2^H_4_] SAG was synthesized similarly to SAG by utilizing [^2^H_4_] MeSA in place of MeSA as starting material and was >99% pure by LC-MS.

Extraction: Frozen plant material was ground in liquid nitrogen to a fine powder using a mortar and pestle and stored at −80 °C until chemical analysis. The samples were weighed (100 mg FW) and to each was added 1 mL chilled (4 °C) extraction solvent (acetonitrile +0.01% TFA), labeled internal standard mix (2.5 ng [^2^H_4_] SA, 25 ng [^2^H_5_] JA, 6.4 ng [^2^H_6_] ABA, 0.6 ng [^2^H_10_] JA-Ile, 5 ng [^2^H_4_] SAG, 25 ng [^13^C_6_] IAA and 10 ng [^2^H_5_] MeJA) and 0.8 g stainless steel beads 0.9–2 mm (Next Advance Inc., NY, USA). Samples were bead beaten for 5 min (Bullet Blender 24 Gold, Next Advance Inc., NY, USA) and were extracted in the dark overnight at 4 °C using an end-over-end rotator at 30 rotations/min. After centrifugation at 16,000× *g* for 5 min, supernatant from each sample was transferred into a well of 96-well collection plate (Phenomenex, CA, USA). The remaining pellet was re-extracted with 0.5 mL of the chilled extraction solvent, combined with the first supernatant, and evaporated to dryness using a CentriVap concentrator (Labconco, Kansas City, MO, USA) at −4 °C. Sample clean-up employed graphitized carbon following a method described by Cai et al. [116] with modifications to adapt to a 96-well plate format. Briefly, samples were reconstituted in chilled (4 °C) 80:20 acetonitrile:water (1 mL) and shaken for 20 min on a flat-bed orbital shaker prior to SPE clean-up on a Hypersep Hypercarb 96-well plate (25 mg/1 mL; Thermo Scientific, CA, USA). Plates were conditioned using 1 mL acetonitrile followed by 1 mL water. After conditioning, samples were loaded and then the acidic phytohormones were eluted with 500 μL of acetonitrile and evaporated to dryness in a refrigerated centrivap vacuum concentrator at −4 °C. Samples were reconstituted in 200 μL of 10:90 acetonitrile:water for analysis by LC-MS.

LC-MS Analysis: LC-MS/MS experiments were performed on a 5500 QTrap triple quadrupole/linear ion trap (QqLIT) mass spectrometer equipped with a TurboIon-Spray™ interface (AB Sciex, ON, Canada) coupled to a Shimadzu Exion UHPLC (Kyoto, Japan). Phytohormones were separated on a Poroshell 120 SB-C18 2.7 μm 2.1 mm × 150 mm ID column (Agilent Technologies, CA, USA) maintained at 60 °C. Solvents were (A) water + 0.1% formic acid and (B) acetonitrile + 0.1% formic acid and the flow rate was 400 μL^−1^. The initial mobile phase, 2% B was held for 3 min before ramping linearly to 16% B at 3.5 min, then to 100% B at 7 min and holding at 100% B until 8 min before resetting to the original conditions. Injection size was 10 μL. MS data were acquired in the negative mode, and positive mode (IAA only), using a MRM method with optimized Q1 and Q3 transitions for each of the analyzed acidic phytohormones (Table S3). Other operating parameters were as follows: dwell time, 10 ms; ionspray voltage, −4500 V; ionspray voltage (IAA and MeJA), 4500 V; temperature, 600 °C; curtain gas, 45 psi; ion source gas 1, 60 psi; ion source gas 2, 60 psi. All data were analyzed and processed using Analyst version 1.7.2 and SciexOS version 2.0 software packages. Concentrations were calculated on the basis of the peak area for the endogenous compounds relative to those determined for the internal standards.

## 5. Conclusions

Our findings suggest that elevated AsA confers increased abiotic stress tolerance by increasing ABA and its specifically active catabolite PA. Changes in these in turn activate a complex of stress-response master regulators which have extensive downstream networks that are involved in mediating drought, cold and salt stress responses. Future research should be directed at exploring these AsA-related regulatory networks more completely, identifying potential AsA-related response element(s) in the *NCED3* promoter, evaluating the relative importance of subcellular organelle AsA concentrations and what effects changes in those can have, and further investigating the crosstalk with phytohormones. Clearing up the role of jasmonates also requires more work. Finally, how and where AsA concentration is sensed is another unknown which needs to be further investigated.

## Data Availability

Data is presented in manuscript and in supplementary materials.

## Supplementary Materials

Supplementary materials can be found at https://www.mdpi.com/article/10.3390/ijms22136743/s1.
