# Supplementary material for: Elevating Ascorbate in Arabidopsis Stimulates the Production of Abscisic Acid, Phaseic Acid, and to a Lesser Extent Auxin (IAA) and Jasmonates, Resulting in Increased Expression of DHAR1 and Multiple Transcription Factors Associated with Abiotic Stress Tolerance
Source: Int J Mol Sci. 2021 Jun 23;22(13):6743. doi: 10.3390/ijms22136743 (PMC8269344; doi:10.3390/ijms22136743)
Supplement: Supplementary file 1 [file ijms-22-06743-s001.zip › Supplementary figure 1.pdf]

Elevating ascorbate in Arabidopsis stimulates the production of abscisic acid, phaseic acid, and to a lesser auxin (IAA) and jasmonates, resulting in increased expression of DHAR1 and multiple transcription factors associated with abiotic stress tolerance.

Sean M. Bulley, Janine M. Cooney, and William Laing

Supplementary data

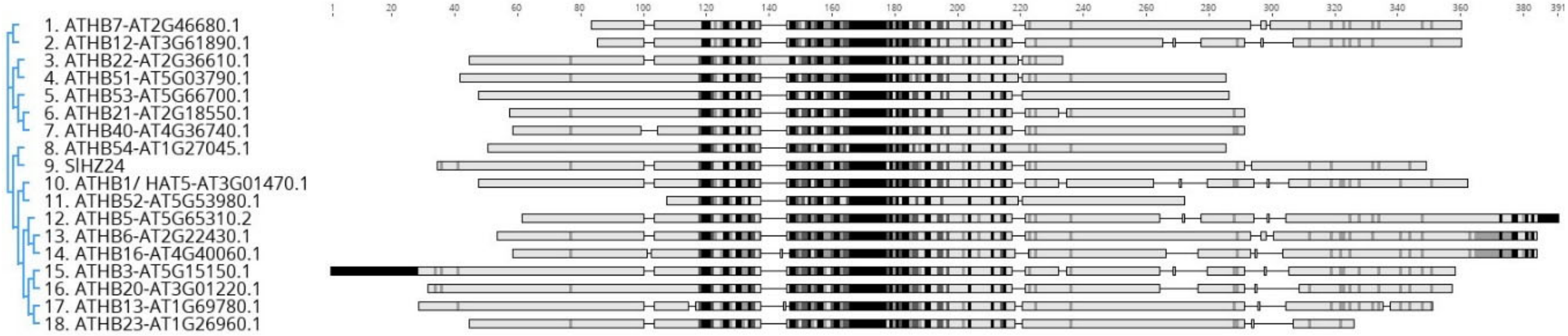

Supplementary Figure S1: Arabidopsis protein alignments with SlHZ24. Genes where expression was measured are numbered 1, 2, 8, 10, 11, 13, and 14 in figure. Alignment tree generated using Geneious version 10.0 (Biomatters; http://www.geneious.com).
